# CAN-DAQ: An open-source, cost-effective data capture device and software for automotive research

**DOI:** 10.1016/j.ohx.2026.e00743

**Published:** 2026-02-02

**Authors:** Anuj Verma, Chandram Millon Dutta, Aritra Ghosh, Sakshin M. Kanchibail, Sneha Harish, Rishvanth S.K., Shaurya Chandra, Siddharth Das, Selvakumar K.

**Affiliations:** Vellore Institute of Technology, Tiruvalam Rd, Katpadi, Vellore, Tamil Nadu 632014, India

**Keywords:** Automotive, Controller Area Network (CAN), Data acquisition (DAQ), CAN database (DBC), Real-time graphing, Database management

## Abstract

Modern systems, from vehicles to industrial testbenches, generate vast amounts of CAN bus data, yet researchers and developers lack affordable, open-source tools for its capture and analysis. While commercial tools are cost-prohibitive and existing open-source options often lack integrated hardware or mature software, acquiring this data is essential for subsystem validation (such as powertrains, safety systems, and sensors), ECU development, and network security analysis, with real-time graphing providing immediate insight. We present CAN-DAQ, a complete hardware–software platform that bridges this gap, matching the core features of commercial systems at a fraction of the cost. It combines an ESP32-based hardware interface with a flexible Python-based software and SDK, featuring high-resolution real-time visualization and a robust SQL backend. CAN-DAQ supports all classic CAN baud rates from 25 kbps to 1 Mbps and achieves a maximum sampling frequency of 1 kHz, reliably capturing 1000 CAN frames per second. As a fully open-source solution, it provides a foundation for users to build custom real-time data analytics applications. The system’s effectiveness was validated through comprehensive testing of its data reception, transmission, and sampling capabilities, demonstrating reliable operation against commercial-grade automotive CAN interfaces.

## Graphical abstract

**Figure:**
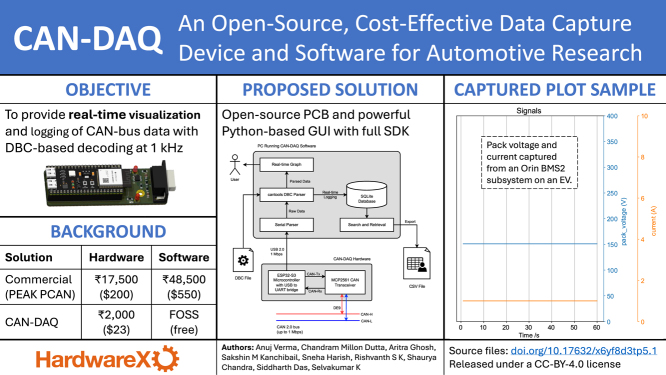

## Specifications table

**Table:** 

Hardware name	CAN-DAQ
Subject area	Educational tools and open source alternatives to existing infrastructure
Hardware type	Field measurements and sensors
Closest commercial analog	PEAK System PCAN-USB
Open source license	CC-BY-4.0
Cost of hardware	INR 2000 (USD 23)
Source file repository	http://doi.org/10.17632/x6yf8d3tp5.1

## 1 Hardware in context

The Controller Area Network (CAN) protocol serves as the backbone of communication in modern vehicular and industrial systems, providing a robust, efficient means of data exchange between electronic control units (ECUs) [1], [2]. Because this bus carries critical operational data, the ability to log, decode, and visualize CAN traffic has become a fundamental requirement for diverse research fields ranging from agricultural automation to electric vehicle (EV) efficiency. While the research community has established sophisticated methodologies for analyzing this data, and numerous technical tools exist to interface with the network, the hardware landscape remains polarized. This paper introduces a platform designed to bridge the divide between expensive, proprietary industrial tools and fragmented open-source alternatives, offering a cohesive hardware–software ecosystem for accessible automotive research.

The utility of CAN data is particularly evident in heavy machinery and efficiency studies, where real-time logging informs design and maintenance strategies. In agricultural sectors, Rohrer et al. demonstrated that custom CAN interface tools allow for real-time monitoring of tractor performance, facilitating in-cab decision-making [3]. Building on this, Götz et al. utilized extensive CAN logging to derive load cycles from tractors, generating a dataset essential for designing the powertrains of future electrified agricultural machinery [4]. The accuracy of such data is paramount; previous evaluations have validated that machine-reported CAN parameters, such as engine torque and speed, correlate strongly with dynamometer measurements, justifying their use in high-fidelity research [5]. Beyond agriculture, CAN data is critical for EV energy management. Lee et al. integrated CAN data with ambient conditions to evaluate energy consumption in passenger EVs, revealing significant efficiency variances based on temperature and HVAC usage [6].

To manipulate and interpret this raw data, the engineering community has developed specialized techniques and software tools. Security researchers, for instance, have introduced systems like X-CANIDS, which parses payloads into human-understandable signals to detect intrusions and zero-day attacks [7]. Similarly, reverse engineering pipelines such as CAN-D have been proposed to automatically decode proprietary signal boundaries and endianness, unlocking interpretability for undocumented vehicles [8]. In the open-source domain, software-centric tools like SavvyCAN have set the standard for visualizing these signals [9], while the Open Vehicle Monitoring System (OVMS) provides a framework for remote monitoring and telematics [10]. These tools underscore a shift toward sophisticated, signal-level analysis that requires reliable, high-frequency data ingestion.

Despite these software advancements, the physical acquisition of data relies on a hardware ecosystem that is currently divided into distinct categories. On one end are high-cost, commercial solutions from vendors like Vector, National Instruments, Peak-System, and CSS Electronics [11], [12], [13], [14], [15]. Even purely software solutions like MATLAB CAN Explorer [16] require expensive licenses, and are often paired with proprietary hardware. These tools offer robust performance, integrated plotting software [17], and reliable support, but their high cost and closed source nature create barriers for academic use-cases. On the other end are academic proofs-of-concept and low-cost alternatives. Researchers have developed various custom loggers using Arduino [18], [19], STM32 [20], [21], and Raspberry Pi [22] platforms. Several studies have presented specific implementations for OBD-II logging [23], [24] and general-purpose acquisition [25], [26], often using LabVIEW for monitoring [27]. Marx et al. compared various logging methods, concluding that while low-cost options can be accurate, file formats and processing needs vary significantly [28].

However, a critical research gap remains in the integration of these open-source technologies. While commercial tools provide a seamless “plug-and-play” experience where the hardware and software are designed to work in unison, the open-source landscape is fundamentally fragmented. Excellent open-source hardware projects like candleLight [20] or generic firmware like connectivity [29] act primarily as adapters, lacking their own dedicated analysis software. Conversely, powerful software like SavvyCAN [9] requires users to source and configure supported hardware independently, and tools like BUSMASTER [30] are no longer actively maintained. There is a notable absence of a unified, low-cost platform that provides the complete pipeline: dedicated hardware, optimized firmware, and a native PC-side GUI capable of DBC-based parsing and real-time plotting out of the box. This fragmentation forces researchers to expend significant effort on system integration rather than data analysis.

We present CAN-DAQ to address this specific gap. As detailed in Table 1, CAN-DAQ synthesizes the performance and usability of commercial tools with the accessibility of open-source hardware. Unlike simple USB-to-CAN adapters, CAN-DAQ is a complete instrument; it combines an ESP32-S3 based hardware interface with a custom, feature-rich software suite. This integration allows for features typically reserved for expensive commercial units, such as automatic signal interpretation via CAN Database (DBC) files, high-resolution real-time plotting, and SQL-backed data logging. By providing a unified, open-source hardware and software stack, CAN-DAQ eliminates the vendor lock-in of commercial tools and the integration complexity of existing open-source alternatives, democratizing access to professional-grade automotive diagnostics.

**Table 1:** Comparison of CAN data logging and analysis solutions.

Solution	Hardware	Software	DBC	Plotting	DE9 Port	Cost
MATLAB CAN Explorer [16]^a^	N/A	Application	Yes	Yes	N/A	USD 2150 (INR 190,000)^c^
VN1610 (Vector) [14]^a^	Ready-made	Firmware, Application	Yes	Yes	Yes	USD 2000 (INR 177,104)^c^
C-DAQ 9862 (NI) [15]^a^	Ready-made	Firmware, Application	Yes	Yes	Yes	USD 1299 (INR 115,000)^c^
PCAN-USB (PEAK) [11], [17]^a^	Ready-made	Firmware, Application	Yes	Yes	Yes	EUR 735 (INR 75,000)
CL2000 (CSS) [12]^a^	Ready-made	Firmware, Application	Yes	Yes	Yes	EUR 230 (INR 23,000)
OBD2-SmartCard-LCD [26]^b^	DIY	No	No	No	No	USD 85 (INR 7500)
PICAN2 [22]^a^	Ready-made	Firmware	No	No	Yes	GBP 63 (INR 6500)
usb2can [21]^b^	DIY	Firmware	N/A	N/A	No	USD 30 (INR 2500)^d^
candleLight [20]^b^	DIY	No	No	No	Yes	USD 10 (INR 850)^d^
BUSMASTER [30]^b^	N/A	Application	Yes	Yes	N/A	N/A (Open-source)
SavvyCAN [9]^b^	N/A	Application	Yes	Yes	N/A	N/A (Open-source)
Serial_CAN_Arduino [19]^b^	N/A	Firmware	N/A	N/A	N/A	N/A (Open-source)
cannectivity [29]^b^	N/A	Firmware	N/A	N/A	N/A	N/A (Open-source)
**CAN-DAQ (Ours)**^b^	**DIY**	**Firmware, Application**	**Yes**	**Yes**	**Yes**	**USD 23 (INR 2000)**

a Closed-source.

b Open-source.

c Our estimates based on third-party retailers, since an official quote is not available on the website.

d Our estimates based on the Bill of Materials (BOM) provided by the maintainers, since they have not provided a total cost.

## 2 Hardware description

The ESP32-S3 [31] was chosen as the main microcontroller for its robust performance and future-proof upgrade path to RISC-V options like the ESP32-Cx series. Modern devices, like the CANedge2 from CSS Electronics which streams data over Wi-Fi [13], show that connectivity options such as USB-OTG, Wi-Fi, and Bluetooth are becoming increasingly important for applications like wireless data streaming and in-vehicle analytics. These features are readily available on the ESP32-S3 [32]. Communication with the CAN bus is achieved using an MCP2561 CAN transceiver [33], preferred over the obsolete MCP2551 due to its simpler upgrade path to the MCP2561FD [34], and proven reliability. The transceiver is integrated with the ESP32-S3 to support CAN 2.0 communication at standard baud rates.

**Fig. 1:**
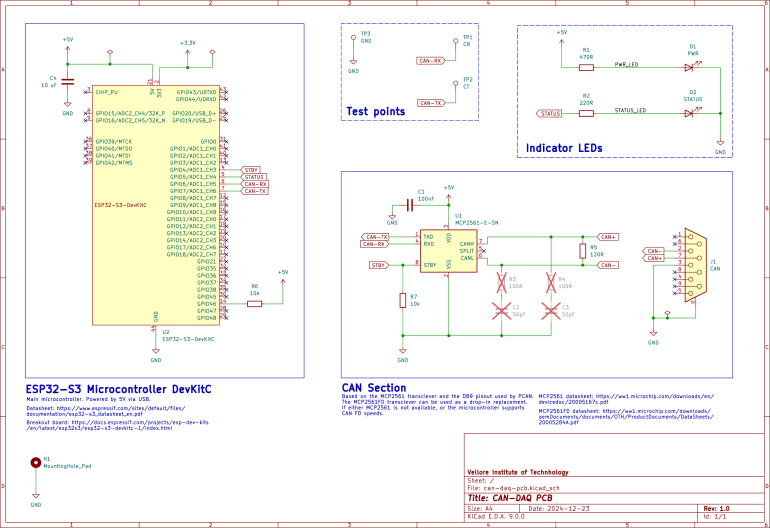
Schematic of the complete CAN-DAQ PCB.

The design uses the ESP32-S3’s built-in USB-to-UART interface, accessible via a micro-B connector, for reliable communication with a host PC at up to 3 Mbps. In addition, the hardware design mitigates potential data integrity issues by disabling the ESP32’s debug log output using a pull-up resistor on GPIO46 [35]. A 120 Ω termination resistor is soldered between the CANH and CANL lines to ensure proper signal termination and minimize reflections on the CAN bus. The MCP2561 transceiver features a standby (STBY) pin that is not actively used by the software; it is therefore fixed by connecting it to ground through a 10 kΩ resistor, while also being routed to GPIO4 of the ESP32-S3 for potential future customization. This design is implemented on a compact printed circuit board (PCB) developed in KiCAD, as shown in Fig. 1.

The embedded firmware, developed in C++ using the ESP-IDF framework [36], is responsible for configuring the TWAI (Two-Wire Automotive Interface) of the ESP32-S3, managing real-time CAN data acquisition, and handling USB communication. The current implementation relies on the ESP32’s TWAI driver (ESP-IDF HAL), which automatically filters and discards CAN error frames at the hardware level. This ensures that only valid data is logged; while these discarded frames are not currently reported to the end-user, this reporting feature may be incorporated in future software versions.

**Fig. 2:**
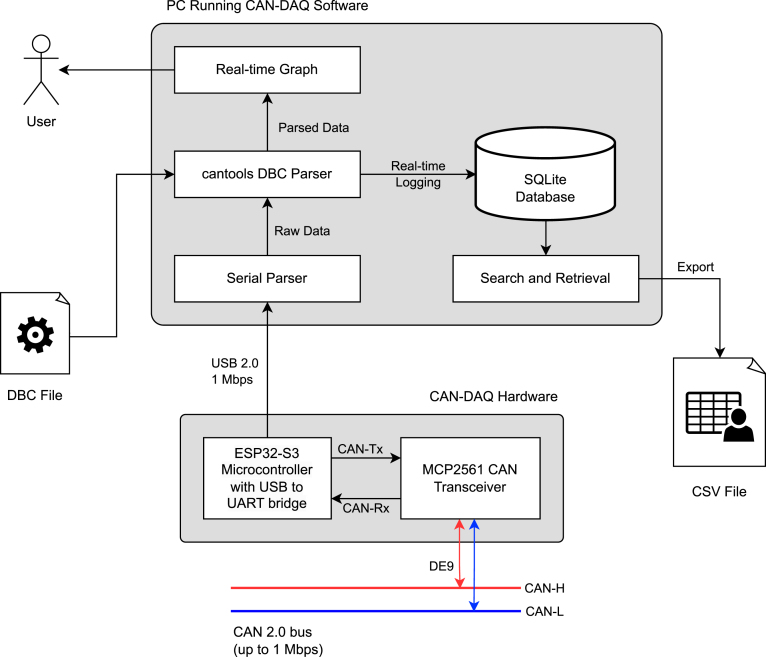
System architecture.

**Fig. 3:**
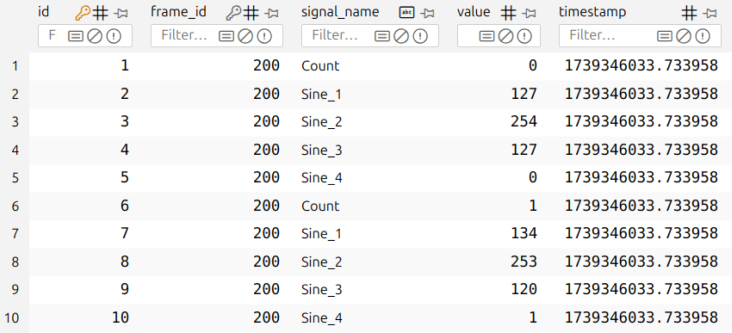
Screenshot showing a few rows of logged CAN signals in the SQLite database.

In parallel, the PC-side GUI is implemented in Python, using Tkinter for the interface and Matplotlib for real-time plotting. PySerial [37] is used to acquire the raw data over UART and cantools [38] is used for parsing CAN messages against user-supplied DBC files (Fig. 2). Beyond visualization, the software manages high-performance data logging by storing decoded signals in an SQLite3 database as shown in Fig. 3. To ensure reliability at high data rates, records are committed in user-configurable batches using a relational table structure. The software abstracts properties inferred from the DBC file into dedicated Signal and Message data structures, and these are later used by cantools to decode incoming CAN frames. The acquisition of raw data is deliberately decoupled from the decoding process to facilitate custom analytics at any stage of the data pipeline.

## 3 Design files

See Table 2.

**Table 2:** Design files.

Design filename	File type	Open source license	Location of the file
README.pdf	PDF	CC BY 4.0	Mendeley Database
can-daq-pcb.kicad_pcb	KiCAD PCB Layout File	CC BY 4.0	Mendeley Database
can-daq-pcb.kicad_prl	KiCAD PCB Project Related Files	CC BY 4.0	Mendeley Database
can-daq-pcb.kicad_pro	KiCAD PCB Project File	CC BY 4.0	Mendeley Database
can-daq-pcb.kicad_sch	KiCAD PCB Schematic File	CC BY 4.0	Mendeley Database
gerbers.zip	ZIP	CC BY 4.0	Mendeley Database
can-daq-bom.csv	CSV	CC BY 4.0	Mendeley Database
can-daq-pcb-all-pos.csv	CSV	CC BY 4.0	Mendeley Database
can-daq.step	STEP	CC BY 4.0	Mendeley Database
case-design.step	STEP	CC BY 4.0	Mendeley Database
case-upper.stl	STL	CC BY 4.0	Mendeley Database
case-lower.stl	STL	CC BY 4.0	Mendeley Database
can-daq-idf-firmware	ZIP	CC BY 4.0	Mendeley Database
can-daq-application	ZIP	CC BY 4.0	Mendeley Database
can-daq-windows-x64.exe	Executable	CC BY 4.0	Mendeley Database
can-daq-linux-x64	Executable	CC BY 4.0	Mendeley Database
can-daq-macos-x64	Executable	CC BY 4.0	Mendeley Database
manual.pdf	PDF	CC BY 4.0	Mendeley Database

## 4 Bill of materials summary

The 3D printed case was FDM printed on a personal 3D printer, using PLA at 30% infill (see Table 4). Our PCBs were printed by PCB Power and components were hand-soldered. However, we include both a BOM and pick-and-place files along with the designs (see Table 3).

**Table 3:** Bill of materials.

Designator	Component	Number	Cost per unit	Total cost	Source of materials	Material type
PCB1	Main PCB	1	407.00 INR	407.00 INR	Fabricated by PCB Power, designs included	Electronics
U1	ESP32-S3 DevKitC module	1	1251.00 INR	1251.00 INR	https://robu.in/product/espressif-esp32-s3-devkitc-1-n8-developmnt-board/	Electronics
U2	MCP2561 CAN transceiver	1	124.00 INR	124.00 INR	https://robu.in/product/mcp2561-e-p-microchip-can-interface-transceiver-can-transceiver-1-mbps-4-5-v-5-5-v-pdip-8-pins/	Semiconductor
C1	Capacitor SMD 0805 100 nF	1	0.57 INR	0.57 INR	https://robu.in/product/im05b104k500nt-fh-smd-multilayer-ceramic-capacitor-0-1-µf-100-nf-50-v-0805-2012-metric-±-10-x7r/	Semiconductor
C4	Capacitor SMD 1206 10 μF	1	6 INR	6 INR	https://robu.in/product/1206b106k160nt-fh-16v-10uf-x7r±10-1206-multilayer-ceramic-capacitors-mlcc-smd-smt-rohs/	Semiconductor
R1	Resistor SMD 1206 470R	1	0.65 INR	0.65 INR	https://robu.in/product/470-ohm-1-4w-1206-surface-mount-chip-resistor-pack-of-100/	Semiconductor
R2	Resistor SMD 1206 220R	1	1 INR	1 INR	https://robu.in/product/yageo-220-ohm-1-4w-1206-surface-mount-resistor-pack-of-50/	Semiconductor
R5	Resistor SMD 1206 120R	1	0.50 INR	0.50 INR	https://robu.in/product/rc1206fr-07120rl-yageo-res-thick-film-1206-120-ohm-1-0-25w1-4w-±100ppm-c-pad-smd-t-r/	Semiconductor
R6, R7	Resistor SMD 1206 10k	2	0.69 INR	1.39 INR	https://robu.in/product/10k-ohm-1-4w-1206-surface-mount-chip-resistor-pack-of-100/	Semiconductor
D1	LED THT 3 mm red	1	1.30 INR	1.30 INR	https://robu.in/product/3mm-red-dip-led-pack-of-50/	Semiconductor
D2	LED THT 3 mm blue	1	1.58 INR	1.58 INR	https://robu.in/product/3mm-blue-dip-led-pack-of-50/	Semiconductor
J1	DE9 horizontal female connector	1	23.80 INR	23.80 INR	https://robu.in/product/db9-female-right-angle-connector-pack-of-5/	Non-specific
J2, J3	1 × 22 2.54 mm female header	2	INR 10	INR 20	https://robu.in/product/2-54mm-1x40-pin-female-single-row-header-strip-pack-of-10/	Non-specific
CASE1	3D printed case	1	50 INR	50 INR	Printed from PLA on a personal printer, design files included	Inorganic
SCREW1	M2.5 screws	2	1 INR	2 INR	Local hardware store	Metal

## 5 Build instructions

### 5.1 Manufacturing and assembling the CAN-DAQ PCB

**Fig. 4:**
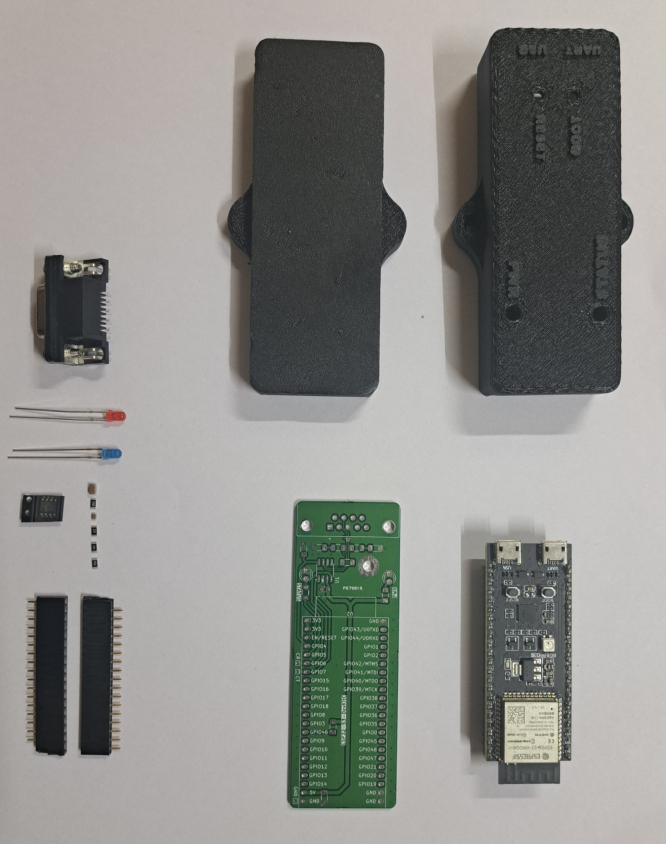
Bare components.

Our design is centered around a printed circuit board (PCB), on to which the MCP2561 transceiver is soldered and the ESP32-S3 DevKitC microcontroller breakout board is mounted. The provided Gerber files may be sent to any local PCB manufacturer to print a bare PCB, on to which components may be hand-soldered. Additionally, a BOM with manufacturer part numbers (MPN), and pick-and-place file, are also provided that can be used to order the PCBs factory-assembled. The bare parts are shown in Fig. 4 and assembled PCB (without the ESP32 microcontroller mounted) is shown in Fig. 5(a).

The system, as shown in Fig. 5(b), can be used as-is, provided the firmware is correctly flashed using the ESP32-IDF software [36]. However, if the bottom of the PCB comes into contact with a conductive surface (such as a metallic table), short-circuits may be created which can damage the device. Therefore, the files required to 3D-print and assemble a protective plastic casing are also provided.

Fig. 5Photograph of assembled CAN-DAQ PCB.Fig. 5

**(a):**
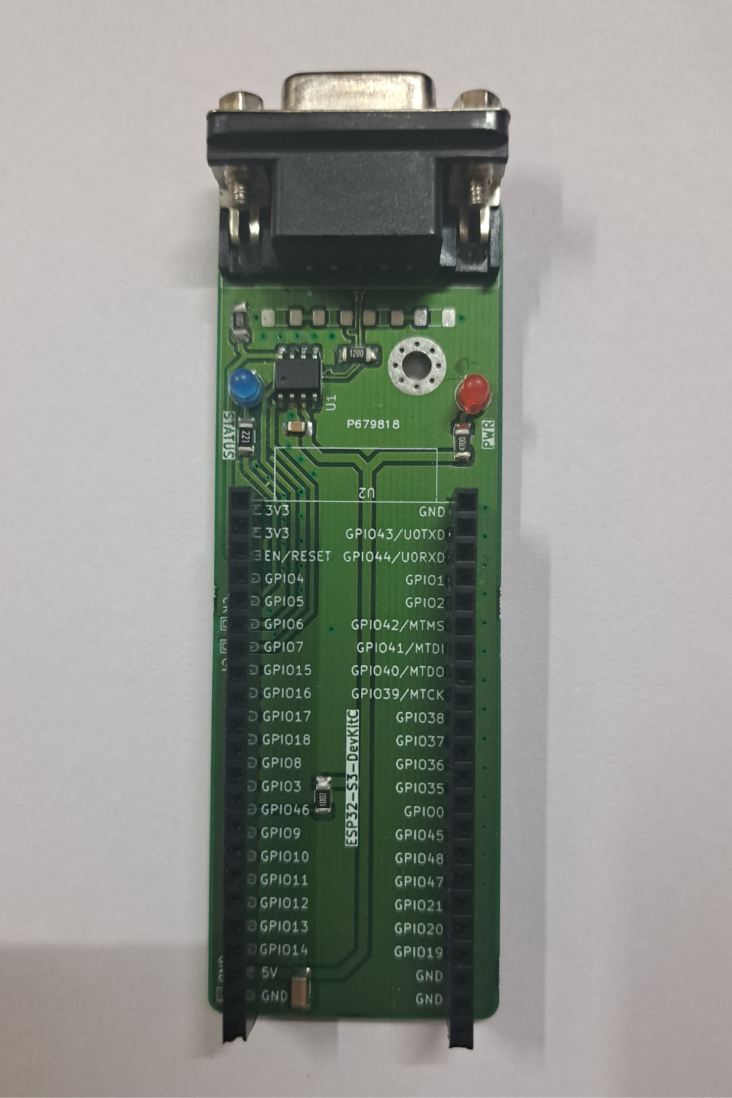
Without ESP32.

**(b):**
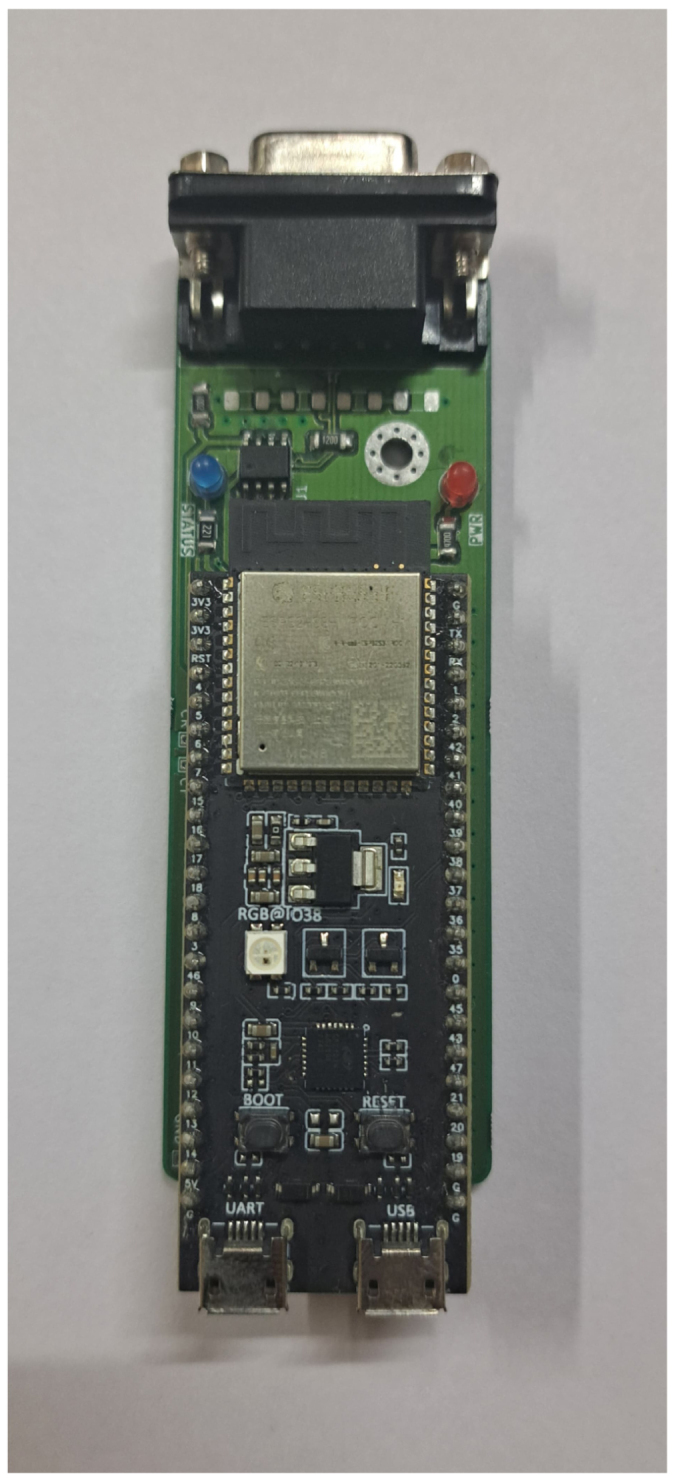
With ESP32.

### 5.2 Downloading and printing 3D components

**Fig. 6:**
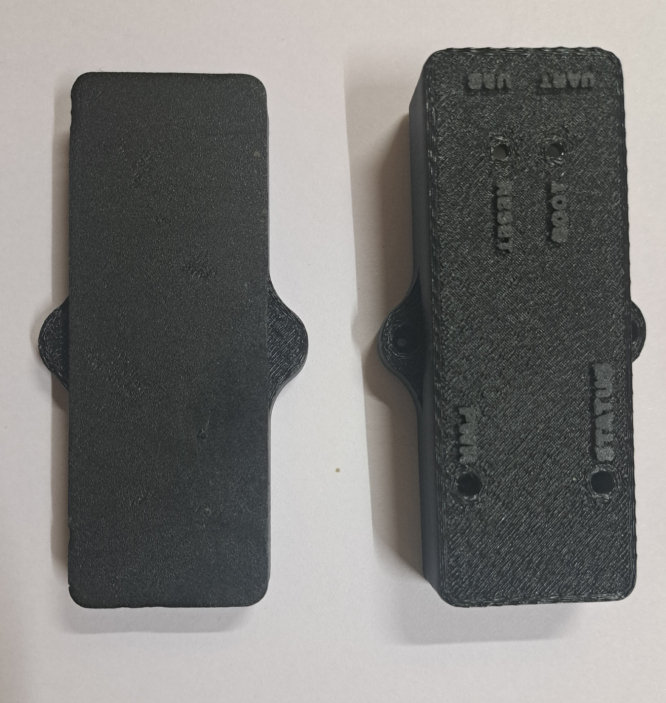
3D printed enclosure (front and back).

The case is a two-part assembly, composed of a bottom housing and a top-cover. The top cover has open holes for LEDs and connectors, and labels to indicate their function. The CAD design is provided as STL files for easily printing directly, as well as a STEP file in case any modifications are desired. The finished halves of the enclosure is shown in Fig. 6. While our model was sliced using Cura, any slicing tool may be used. We used the configurations shown in Table 4 to print the case using black polylactic acid (PLA) filament using fused deposition modeling (FDM).

**Fig. 7:**
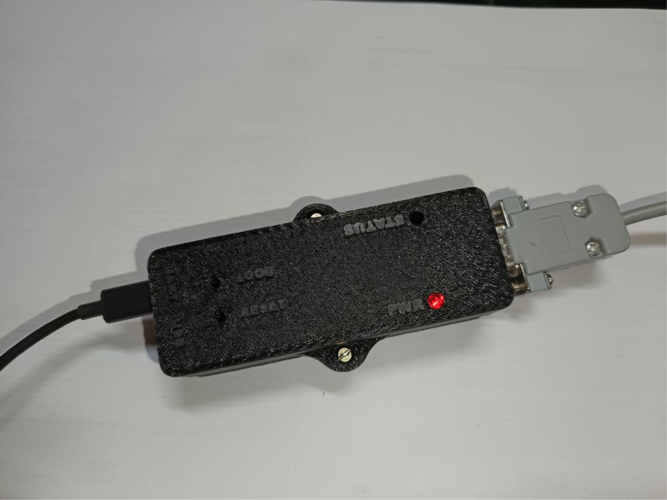
CAN-DAQ connected and powered ON.

The final assembly of the CAN-DAQ system involves the integration of the PCB and its peripheral components into the custom-designed enclosure. Initially, the lower half of the case is positioned with its flat side oriented downward to serve as a stable base. The assembled PCB, including the ESP32 microcontroller breakout board, is then carefully inserted, ensuring precise alignment of all external connectors and interfaces. Subsequently, the upper half of the case is aligned and placed over the assembly, with particular attention to the correct positioning of status indicators and ports. The enclosure is secured with two M2.5 bolts to ensure mechanical stability and reliable operation. The completed assembly is depicted in Fig. 7.

**Table 4:** 3D printing configurations.

Property	Value
Support structure	Tree
Support placement	Everywhere
Support overhang angle	59°
Build plate adhesion type	Brim
Infill density	30%
Infill pattern	Gyroid
Printing temperature	200°C
Build plate temperature	60°C
Print speed	180 mm/s

## 6 Operation instructions

### 6.1 Connecting to CAN-DAQ

The user should begin by connecting the CAN-DAQ hardware to both the target CAN bus and the computer. The CAN bus is interfaced using a standard DE9/DB9 connector [39], while CAN-DAQ is connected to the computer via a micro-B USB cable through the “UART” port. Upon connection, the red “PWR” LED will illuminate, indicating that the device is powered on, as shown in Fig. 7. It is necessary to flash the firmware using the ESP32-IDF environment [36], and users may need to install additional driver software for proper communication with the USB-to-UART bridge on the ESP32 breakout board, as indicated in the relevant datasheet.

### 6.2 Using the CAN-DAQ software

**Fig. 8:**
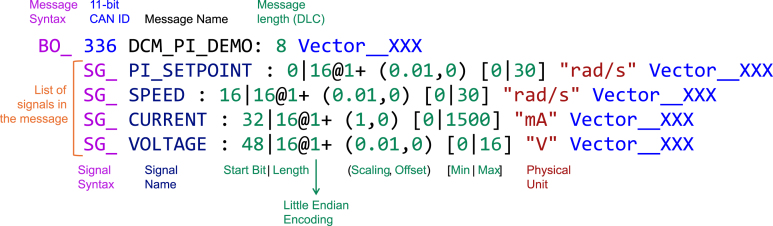
Syntax of an entry in a DBC file.

**Fig. 9:**
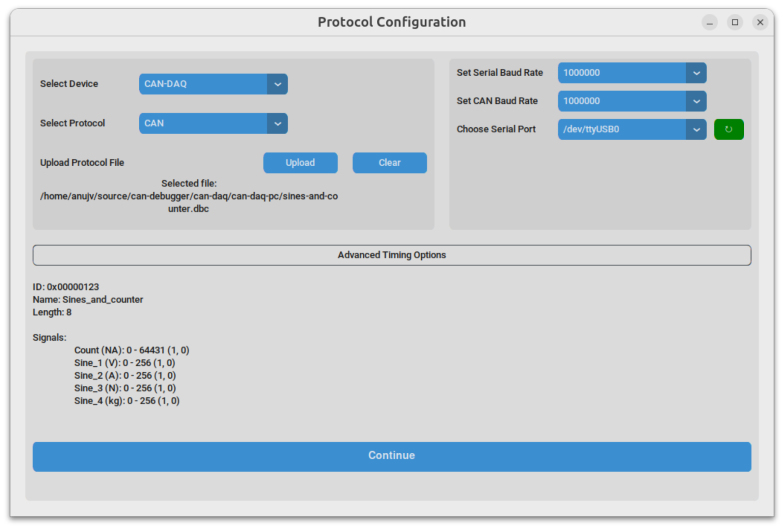
Session configuration screen.

The software interface, which is available for Windows, macOS, and Linux, is distributed as a standalone executable. When first launched, the application guides the user through a series of setup wizards designed to configure a data logging session. In the session management screen, the user can either create a new logging session — thereby defining the destination database for collected data — or retrieve and export a previous session as CSV. The subsequent protocol configuration screen allows for the specification of the CAN protocol by providing a CAN database container (DBC) file, which defines the structure and interpretation of incoming messages (see Fig. 8). The DBC file can be generated using freely available third-party tools such as Vector CANdb++ or the CSS Electronics DBC Editor [40], [41], [42]. In addition to configuring the protocol, users must select appropriate baud rates and assign the relevant communication ports; note that the baud rate for the USB/UART communication is set to 1 Mbps by default in our firmware, while the baud rate for the CAN bus is configured separately. A representative screenshot of the protocol configuration interface is provided in Fig. 9.

**Fig. 10:**
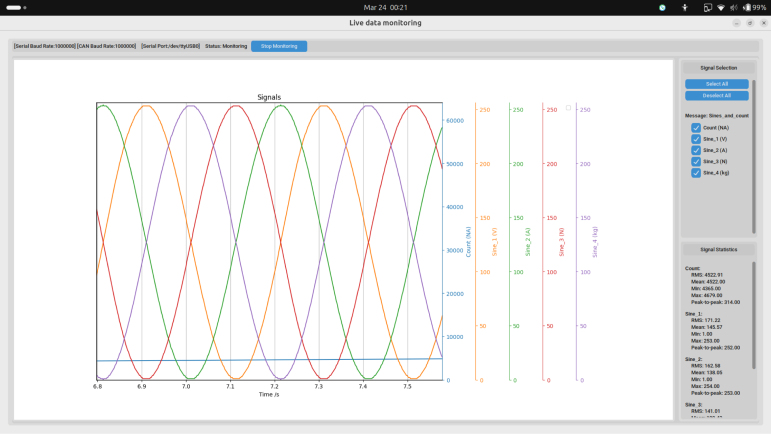
Monitoring screen.

Once configured, the monitoring screen provides a live view of the acquired CAN data. The user initiates data logging by clicking the “Start Monitoring” button, which both enables the CAN-DAQ hardware and begins capturing data. During active monitoring, the blue “Status” LED indicates that valid CAN messages are being received. The desired signals — parsed from the provided DBC file — can be selectively visualized and used for statistical analysis via checkboxes available in the interface. Once the “Start Monitoring” button is pressed, all valid CAN messages acquired are continuously logged to the database until “Stop Monitoring” is pressed, regardless of checkbox selection; however, for the data to be visible on the graph, the corresponding signal’s checkbox must be checked. Fig. 9, Fig. 10 illustrate the session configuration and monitoring interface, respectively.

## 7 Validation and characterization

### 7.1 Specifications and capabilities of the device

See Table 5.

**Table 5:** Technical specifications of CAN-DAQ.

Parameter	Specification
CAN standard	CAN 2.0
Maximum CAN baud rate	1 Mbps
Maximum sampling rate	1 kHz (number of CAN frames per second)
Communication interface	USB 2.0 (micro-B connector; up to 3 Mbps)
Operating voltage	5 V (max 500 mA) by USB port
Microcontroller	ESP32-S3 DevKitC1
CAN transceiver	MCP2561 (upgradeable to MCP2561FD)
Software	Embedded firmware (C++ with ESP-IDF) and PC GUI (Python, Tkinter, matplotlib)

### 7.2 Validation of CAN transmission speeds

**Fig. 11:**
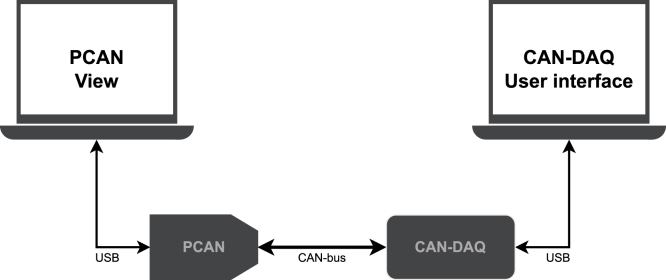
Test setup with PCAN-USB to validate CAN transmission and reception.

To verify the CAN-DAQ system’s performance, tests were conducted to assess both its reception and transmission capabilities with typical CAN bus configurations (see Fig. 11). In the reception test, the system successfully captured data transmitted by a commercial PCAN-USB interface [11] across the full range of standard CAN baud rates, with the acquired values matching those stored in the database. This demonstrates reliable real-time operation across different CAN baud rates. In the transmission test, a MATLAB-based PCAN setup was configured as a CAN server. Operating as a client, CAN-DAQ transmitted a series of messages and correctly received responses for the expected messages while ignoring out-of-scope transmissions. These results confirm that the CAN-DAQ system reliably handles both transmission and reception of CAN messages, enabling it to interactively request data from devices that require polling.

### 7.3 Sampling frequency

To validate the maximum sampling frequency of 1 kHz, a controlled data acquisition test was conducted using a reference STM32 system that transmitted a continuous stream of CAN messages. CAN-DAQ successfully captured and logged all incoming messages at a rate of 1000 full messages per second without dropping any CAN frames, as confirmed by analysis of the recorded data. This result demonstrates the device’s capability for reliable real-time data acquisition at the specified sampling frequency, as illustrated in Fig. 10.

### 7.4 Real-world demonstration

A live test was conducted by connecting CAN-DAQ to the CAN bus used by Orion BMS2 [43], a commercially available battery management system (BMS). The BMS is a subsystem of the electric vehicle developed by a university team. In this test, the BMS operated at a baud rate of 250 kbps and transmitted data at a 2 Hz sampling rate, and according to its documentation, pack voltage and current are broadcast using the 29-bit (extended) CAN identifier 0x1806E9F4 [44], [45]. CAN-DAQ successfully logged messages with the CAN ID 0x1806E9F4, including a pack voltage of 151 V and a pack current of 1 A, all of which matched the expected values. Figs. 12(a) and 12(b) illustrate the experimental setup and data visualization, respectively.

Fig. 12Real-world validation with Orion BMS2.Fig. 12

**(a):**
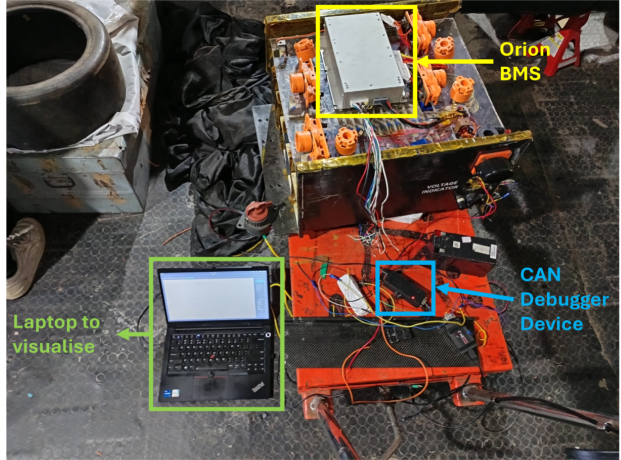
Experimental setup.

**(b):**
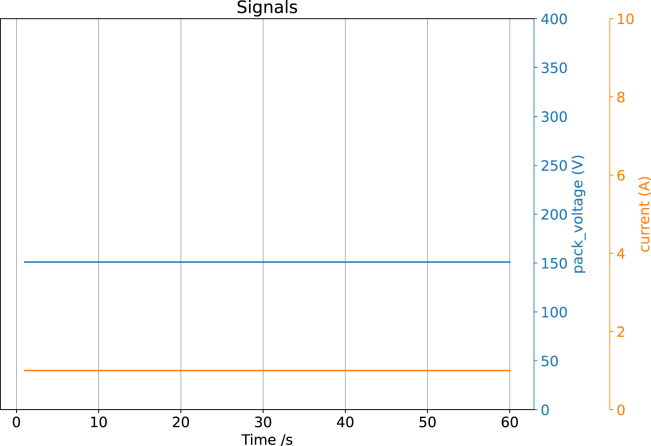
Acquired data plotted on our graph.

### 7.5 Utility in education

To further demonstrate the utility of CAN-DAQ, the system was integrated with a custom DC Motor Development Board for control systems education, using a Quanser Servo Motor [46], [47] as the actuator. The board, which features an STM32F4 microcontroller and supports 1 Mbps CAN communication, served as an ideal test platform. CAN-DAQ was connected to the motor control board via the CAN bus and logged 500 samples per second while the onboard PI controller stabilized the motor speed. Although the resulting graph appears somewhat noisy, it reflects the high-resolution data capture of every minute detail in the controller’s performance. A photograph of the setup is shown in Fig. 13(a) and a screenshot of the data visualization is provided in Fig. 13(b).

Fig. 13Test with the DC Motor Board.Fig. 13

**(a):**
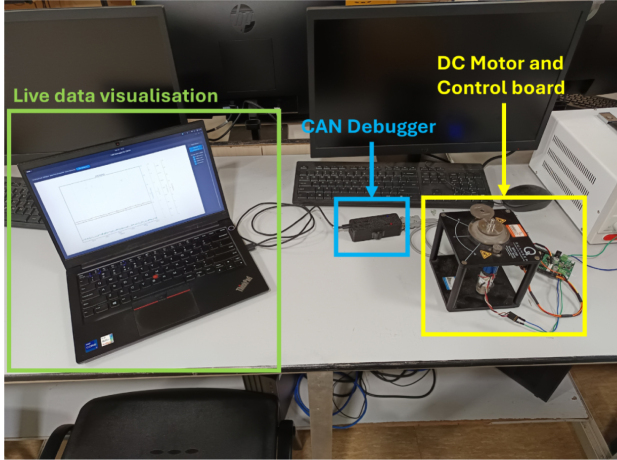
Experimental setup.

**(b):**
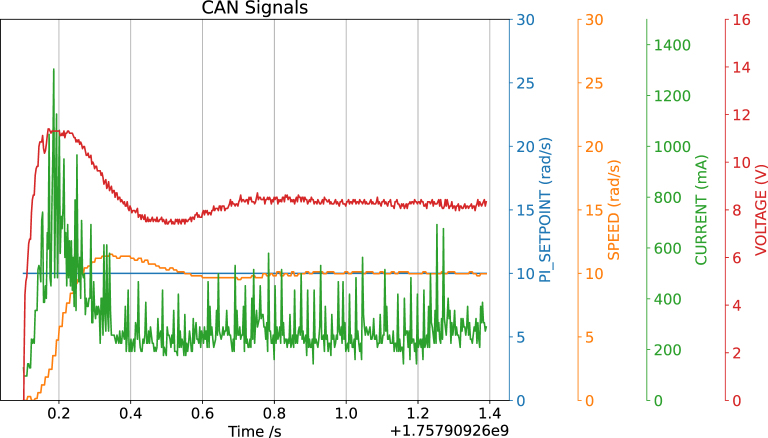
Motor settling after giving a step input with the DC Motor Board.

## CRediT authorship contribution statement

**Anuj Verma:** Writing – original draft, Validation, Software, Project administration, Methodology. **Chandram Millon Dutta:** Software. **Aritra Ghosh:** Software, Investigation. **Sakshin M. Kanchibail:** Methodology. **Sneha Harish:** Writing – review & editing, Investigation, Conceptualization. **Rishvanth S.K.:** Methodology, Conceptualization. **Shaurya Chandra:** Software, Conceptualization. **Siddharth Das:** Validation. **Selvakumar K.:** Writing – review & editing, Resources, Funding acquisition, Conceptualization.

## Declaration of competing interest

The authors declare that they have no known competing financial interests or personal relationships that could have appeared to influence the work reported in this paper.

## References

[1] Corrigan S. (2002). https://www.ti.com/lit/an/sloa101b/sloa101b.pdf.

[2] Robert Bosch GmbH (1991). Bosch Controller Area Network (CAN) Version 2.0 Protocol Standard.

[3] Rohrer R., Pitla S., Luck J. (2019). Tractor CAN bus interface tools and application development for real-time data analysis. Comput. Electron. Agric..

[4] Götz K., Kusuma A., Dörfler A., Lienkamp M. (2025). Agricultural load cycles: Tractor mission profiles from recorded GNSS and CAN bus data. Data Brief.

[5] Rohrer R.A., Luck J.D., Pitla S.K., Hoy R. (2018). Evaluation of the accuracy of machine reported CAN data for engine torque and speed. Trans. ASABE.

[6] Lee G., Song J., Lim Y., Park S. (2024). Energy consumption evaluation of passenger electric vehicle based on ambient temperature under Real-World driving conditions. Energy Convers. Manage..

[7] Jeong S., Lee S., Lee H., Kim H.K. (2024). X-CANIDS: Signal-Aware explainable intrusion detection system for controller area Network-Based In-Vehicle network. IEEE Trans. Veh. Technol..

[8] Verma M.E., Bridges R.A., Sosnowski J.J., Hollifield S.C., Iannacone M.D. (2021). CAN-D: A modular Four-Step pipeline for comprehensively decoding controller area network data. IEEE Trans. Veh. Technol..

[9] Kidder C. (2025). https://github.com/collin80/SavvyCAN/.

[10] Open Vehicles (2025). https://github.com/openvehicles/Open-Vehicle-Monitoring-System-3.

[11] Peak-System Technik GmbH (2025). https://www.peak-system.com/PCAN-USB.199.0.html?L=1.

[12] CSS Electronics (2025). https://www.csselectronics.com/products/can-bus-logger-interface-cl2000.

[13] CSS Electronics (2025). https://www.csselectronics.com/products/can-bus-data-logger-wifi-canedge2.

[14] (2025). https://www.vector.com/us/en/products/products-a-z/hardware/network-interfaces/vn16xx/.

[15] (2024). https://www.ni.com/docs/en-US/bundle/ni-9862-specs/page/specs.html.

[16] The MathWorks, Inc (2025). https://in.mathworks.com/help/vnt/ug/canexplorer-app.html.

[17] Peak-System Technik GmbH (2025). https://www.peak-system.com/PE6-Plotter-Add-in-6.417.0.html?%26L=1.

[18] Yadav A., Sakle N. (2023). Development of low-cost data logger system for capturing transmission parameters of two-wheeler using Arduino. Mater. Today: Proc..

[19] Longan-Labs (2025). https://github.com/Longan-Labs/Serial_CAN_Arduino.

[20] HubertD (2025). https://github.com/HubertD/candleLight.

[21] Matejx (2025). https://github.com/matejx/usb2can.

[22] Copperhill Technologies (2025). https://copperhilltech.com/pican-hats/.

[23] Ramai C., Ramnarine V., Ramharack S., Bahadoorsingh S., Sharma C. (2022). Framework for building Low-Cost OBD-II Data-Logging systems for battery electric vehicles. Vehicles.

[24] Bedretchuk J.P., Arribas García S., Nogiri Igarashi T., Canal R., Wedderhoff Spengler A., Gracioli G. (2023). Low-Cost data acquisition system for automotive electronic control units. Sensors.

[25] Echeverry-Mejia J., Arenas-Uribe F., Contreras D., Vásquez V. (2023). Design and validation of an In-Vehicle data recorder system for testing purposes. IEEE Lat. Am. Trans..

[26] Palomino J., Cuty E., Huanachin A. (2021). 2021 IEEE International Workshop of Electronics, Control, Measurement, Signals and their Application To Mechatronics.

[27] Long C., Zhuo L. (2022). 2022 IEEE 2nd International Conference on Electronic Technology, Communication and Information.

[28] Marx S.E., Luck J.D., Pitla S.K., Hoy R.M. (2016). Comparing various hardware/software solutions and conversion methods for Controller Area Network (CAN) bus data collection. Comput. Electron. Agric..

[29] CANnectivity (2025). https://github.com/CANnectivity/cannectivity.

[30] Rbei-Etas (2025). https://github.com/rbei-etas/busmaster.

[31] Espressif Systems (2023). https://www.espressif.com/sites/default/files/documentation/esp32-s3_datasheet_en.pdf.

[32] Espressif Systems (2025). https://docs.espressif.com/projects/esp-dev-kits/en/latest/esp32s3/esp32-s3-devkitc-1/index.html.

[33] Microchip Technology Inc (2013). https://ww1.microchip.com/downloads/aemDocuments/documents/OTH/ProductDocuments/DataSheets/20005167C.pdf.

[34] Microchip Technology Inc (2014). https://ww1.microchip.com/downloads/aemDocuments/documents/OTH/ProductDocuments/DataSheets/20005284A.pdf.

[35] Espressif Systems (2023). https://www.espressif.com/sites/default/files/documentation/esp32-s3_technical_reference_manual_en.pdf.

[36] Espressif Systems (2025). https://idf.espressif.com/.

[37] Liechti C. (2025). https://github.com/pyserial/pyserial.

[38] Moqvist E. (2025). https://cantools.readthedocs.io/en/latest/.

[39] CAN in Automation e. V. (2025). https://www.can-cia.org/can-knowledge/cia-106-connector-pin-assignment.

[40] CSS Electronics (2024). https://www.csselectronics.com/pages/can-dbc-file-database-intro.

[41] Vector Informatik GmbH (2025). https://www.vector.com/se/en/products/products-a-z/software/candb/.

[42] CSS Electronics (2025). https://www.csselectronics.com/pages/dbc-editor-can-bus-database.

[43] Ewert Energy Systems (2025). https://www.orionbms.com/products/orion-bms-standard/.

[44] Ewert Energy Systems (2018). https://www.orionbms.com/manuals/pdf/orionbms2_operational_manual.pdf.

[45] Ewert Energy Systems (2025). https://www.orionbms.com/manuals/utility_o2/.

[46] Quanser Inc (2020). https://quanserinc.box.com/shared/static/ssyc4up473dt8p5lmfoc37shffaqz1s9.pdf.

[47] Quanser Inc (2024). https://www.quanser.com/products/rotary-servo-base-unit/.

